# *PIK3CA* mutation analysis in Chinese patients with surgically resected cervical cancer

**DOI:** 10.1038/srep14035

**Published:** 2015-09-11

**Authors:** Libing Xiang, Wei Jiang, Jiajia Li, Xuxia Shen, Wentao Yang, Gong Yang, Xiaohua Wu, Huijuan Yang

**Affiliations:** 1 Department of Gynecological Oncology, Fudan University Shanghai Cancer Center, Fudan University, Shanghai, 200032, China; 2 Department of Oncology, Shanghai Medical College, Fudan University, Shanghai, 200032, China; 3 Department of Pathology, Fudan University Shanghai Cancer Center, Fudan University, Shanghai, 200032, China; 4 Cancer Institute, Fudan University Shanghai Cancer Center, Fudan University, Shanghai, 200032, China; 5 Central Laboratory, The Fifth People's Hospital of Shanghai, Fudan University, Shanghai 200240, China

## Abstract

The aim of this study was to evaluate the clinicopathological and prognostic relevance of *PIK3CA* mutations in Chinese patients with surgically resected cervical cancer. *PIK3CA* mutations were screened in 771 cervical cancer specimens using reverse transcription polymerase chain reaction and Sanger sequencing. In total, 13.6% (105 of 771) of patients harbored non-synonymous *PIK3CA* mutations. Patients harboring *PIK3CA* mutations were older than patients with wild-type *PIK3CA* (mean age: 50.7 years vs. 47.0 years, P < 0.01). *PIK3CA* mutations were more commonly observed in postmenopausal patients than in premenopausal patients (19.6% vs. 10.2%, P < 0.01). *PIK3CA* mutations were more common in squamous cell carcinomas than in non-squamous cell tumors (15.3% vs 7.3%, of P < 0.01). The 3-year relapse-free survival was 90.2% for *PIK3CA* mutant patients and 80.9% for PIK3CA wild-type patients (P = 0.03). *PIK3CA* mutation was confirmed as an independent predictor for better treatment outcome in the multivariate analyses (HR = 0.54, 95% CI: 0.29–0.99, P = 0.048). *PIK3CA* mutations were significantly associated with less distant metastases (mutant-type: 8/105, wild-type: 98/666, p = 0.048). Thus, patients with mutant *PIK3CA* had distinct characteristics in age, menopausal status, and histological subtype and have better treatment outcome and less distant metastasis after surgery-based multimodal therapy.

Cervical cancer is the eighth deadliest cancer and is responsible for over 20,000 deaths in China annually^1^. Despite significant improvements in screening, diagnosis and therapy of cervical cancer over the past decade, progress in the management of advanced/recurrent cervical cancer has been underwhelming, as the median overall survival in advanced/recurrent cervical cancer patients remains between 10 and 13 months^2^.

Genes involved in the *PI3K* pathway represent potential therapeutic targets for cancers, and *PIK3CA* mutation status may be useful as a biomarker for targeted therapy of cervical cancer. The frequently mutated and constitutively activated PI3K pathway is involved in the pathogenesis of various human cancers. The *PI3K* pathway is an attractive cancer therapeutic target. *PI3K* pathway inhibitors, such as buparlisib, everolimus and temsirolimus, have demonstrated clinical efficacy in several different cancers^3^,^4^,^5^,^6^. *PIK3CA* is one of the most commonly mutated genes associated with cervical cancer^7^. Most *PIK3CA* mutations found in cervical cancer occur in the helical domain (exon 9), whereas a few *PIK3CA* mutations have been reported to affect the kinase domain (exon 20)^8^. Preclinical and clinical studies have demonstrated that *PIK3CA* mutations may predict tumor response to *PI3K* pathway inhibitors^9^,^10^,^11^,^12^. Therefore, the identification and characterization of cervical cancer patients harboring mutant *PIK3CA* are important for designing clinical trials investigating *PI3K* pathway inhibitors.

However, the clinicopathological characteristics and prognostic impact of *PIK3CA*-mutated cervical cancer have not been well established. It is necessary to conduct *PIK3CA* mutational analyses in a large cohort of cervical cancer patients. Therefore, in this study, we investigated the clinicopathological and prognostic relevance of *PIK3CA* mutations in a large cohort of Chinese patients with surgically resected cervical cancer.

## Results

### *PIK3CA* mutation in cervical cancers

The prevalence of *PIK3CA* mutations in our cohort of 771 patients with FIGO stage IB/IIA cervical cancer was determined by RT-PCR–based direct sequencing. Nonsynonymous *PIK3CA* mutations were found in 105 cases (13.6%), and all of these mutations were base substitutions. Ten mutations occurred in the helical domain (HD), whereas 95 mutations occurred in the kinase domain (KD). No tumors harbored mutations in both the HD and KD. In total, 94.3% (99) of the 105 mutations occurred at previously identified hotspots, including 62 amino acid substitutions at residue 545, 32 amino acid substitutions at residue 542 and 5 amino acid substitutions at residue 1,047. The most common mutations were E545K and E542K, both of which affect exon 9, which were found in 59 and 32 samples, respectively. H1047R mutation, which has been reported to predict an increased response to *PI3K/AKT/mTOR* signaling pathway inhibitors, was found in 4 cases^13^. Several rare nonsynonymous base substitutions were identified in our study, some of which have been reported in the Catalog of Somatic Mutations in Cancer (COSMIC) database. Two mutations identified in our study were not previously reported. One hundred mutations (95.2%) were recognized as activating mutations, whereas the effects of the remaining five mutations are unknown (Table 1).

### Clinicopathological association of *PIK3CA* mutations

The 771 patients in our study comprised 606 (78.6%) patients with squamous cell carcinomas (SCCs), 101 (13.1%) patients with adenocarcinomas (ACs), 44 (5.7%) patients with adenosquamous carcinomas (ASCs) and 20 (2.6%) patients with other rare histopathological subtypes. Two-hundred two (26.2%) patients presented with pelvic or paraaortic lymph node metastasis during pathologic examination of the surgical specimens. Five-hundred forty (70.0%) patients underwent post-operative radiotherapy or chemoradiotherapy for a high-risk for recurrence.

Patient age, menopausal status and histological subtypes were strong predictors of *PIK3CA* mutation status. Patients harboring *PIK3CA* mutations were older than patients with wild-type *PIK3CA* (mean 50.7 vs. 47.0 years; P < 0.01) (Table 2). *PIK3CA* mutations were identified in 23.5% of patients aged ≥60 years, 17.5% of patients aged 50 to 59 years, 10.9% of patients aged 40 to 49 years and 8.5% of patients aged <40 years. This trend was significantly different when compared with *PIK3CA* wild-type cohort (P < 0.01). *PIK3CA* mutations were more commonly observed in postmenopausal patients than in premenopausal patients (19.6% vs. 10.2%, P < 0.01). In addition, *PIK3CA* mutations occurred more frequently in squamous cell carcinomas than in non-squamous cell tumors (15.3% vs. 7.3%, P = 0.01). In this study, *PIK3CA* mutation did not correlate with other clinicopathonogical features, such as clinical FIGO stage, node metastasis, tumor size, myometrial invasion, LVSI and parametrial involvement (Table 2).

### Prognostic role of *PIK3CA* mutation

In the median follow-up of 38 months (range: 1–59 months), 142 patients experienced distant metastases (n = 106) or pelvic recurrence (n = 36). *PIK3CA* mutations were significantly associated with distant metastases (mutant-type: 8/105, wild-type: 98/666, p = 0.048), whereas, the correlation between *PIK3CA* mutation status and pelvic recurrence was not confirmed in this cohort of patients (mutant-type: 3/105, wild-type: 33/666, p = 0.459).

**Figure 1:**
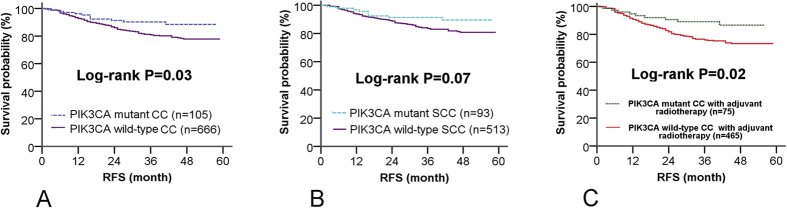
Relapse-free survival (RFS) according to PIK3CA mutation status in patients with cervical cancer (CC) (A), patients with squamous cell cervical cancer (SCC) (B) and patients accepting radiotherapy after surgery (C), respectively.

Univariate analysis revealed a striking association between *PIK3CA* mutations and patient survival. Patients harboring mutant *PIK3CA* showed a better treatment outcome than patients with wild-type *PIK3CA*. The 3-year relapse-free survival (RFS) rate was 90.2% for *PIK3CA* mutant patients and 80.9% for *PIK3CA* wild-type patients (P = 0.03) (Fig. 1A and Table 3). Our multivariate analyses revealed that *PIK3CA* mutation in cervical cancer was an independent predictor for better RFS (HR = 0.54, 95% CI: 0.29–0.99, P = 0.048) (Table 3).

To exclude confounding effects associated with histological subtypes, a survival analysis was conducted in patients with squamous cell carcinomas. A similar dichotomized RFS trend was observed according to *PIK3CA* mutation status in patients with squamous cell carcinomas; however, the difference did not reach statistical significance (3-RFS: 91.3% for mutant *PIK3CA* vs. 83.5% for wild-type *PIK3CA*, P = 0.07), which could be due to the short follow-up duration and the limited number of patients experiencing disease relapse (Fig. 1B). We did not observe enough recurrent cases (2 relapses in 12 cases with *PIK3CA* mutations) to effectively evaluate the association between *PIK3CA* mutation status and RFS in the subgroups of patients with non-squamous cell tumors (shown in supplementary Figure S1A).

A survival association analysis was performed in 540 patients who underwent postoperative radiotherapy. In this group of patients, better treatment outcomes were observed in patients harboring mutant *PIK3CA* than in patients with wild-type *PIK3CA* (3-year RFS: 89.1% vs. 76.0% P = 0.02). In patients who did not receive adjuvant therapy, no significant difference was observed in the 3-year RFS based on the presence or absence of *PIK3CA* mutation (shown in supplementary Figure S1B).

In order to explore which specific mutations in *PIK3CA* would be really useful for prognosis, all the *PIK3CA* mutants were subdivided into three groups according to different mutated position of amino acid residuals (pos.542, pos.545, and other positions). Similar trends of RFS were observed in the three subgroups of patients, while only the subgroup of patients with mutations on pos.542 was confirmed to have better RFS than the patients with wide-type PIK3CA (p = 0.025). The differences of RFS between the patients with mutations on the other positions and those with wide-type did not reach statistical significance probably due to limited cases (p = 0.366 for pos. 545 and p = 0.379 for other positions, supplementary Figure S2A). Furthermore, *PIK3CA* mutations were subdivided according to different exons (exon 9 and exon 20). Similar trends of RFS were observed in the two subgroups. However, only the patients with mutations on exon 9 had better survival than the patients with wide-type (p = 0.040). The difference between the subgroups with mutations on exon 20 and without mutations did not reached statistical significance probably due to limited cases number (p = 0.443, shown in supplementary Figure S2B).

## Discussion

The mutation status in the *PIK3CA* gene could be used as a targeted biomarker for cervical cancer patients. Clinicopathological and prognostic features of *PIK3CA* mutations in tumors remain inconclusive. Although several previous studies have attempted to investigate this association^14^,^15^,^16^,^17^,^18^, most of these studies are underpowered for robust statistical analyses because of small sample size (less than 200 cases), selection bias or a lack of control for confounding factors. Our study is the first large cohort study to investigate the clinicopathological and prognostic relevance of *PIK3CA* mutations in patients with surgically resected cervical cancer.

The prevalence (13.6%) of *PIK3CA* mutations in our study is consistent with that reported in the COSMIC database (10%) as well as with a whole-exome sequencing study (14%)^7^. However, our reported prevalence is also substantially lower than in other investigations (23–33%)^10^,^14^,^15^. The reasons for the differences between these studies and ours may include ethnic heterogeneity, inclusion of late-stage cases and a greater probability of contamination during specimen processing for paraffin-embedded tissues than for fresh tissues. Importantly, our study is consistent with previous investigations in that most of the *PIK3CA* mutations clustered within exon 9. This distribution is distinct in cervical cancers when compared with other types of tumors that present with frequent *PIK3CA* mutations, such as breast cancer^19^ and endometrial cancer^20^. Different mutation hotspots in *PIK3CA* may predict different responses to inhibitors during therapy^13^.

PIK3CA mutations occurred more frequently in older and postmenopausal patients in our study, which is in agreement with a study performed by Cui *et al.*^17^. In addition, the histological subtype can serve as another predictor for *PIK3CA* mutation in cervical cancer. Similar to our results, Alexi *et al.* also observed a higher mutation rate in SCC samples than in AC samples, although, in their study, the difference did not reach statistical significance, which is most likely due to the limited number of cases included in their analyses^14^. No significant differences in the mutation frequencies were observed in tumor subgroups according to other clinical characteristics.

The association between *PIK3CA* mutation status and prognosis was inconclusive. In a study performed by McIntyre *et al.*, *PIK3CA* mutation is associated with a worse overall survival in patients with FIGO stage IB/II cervical cancer treated with radical chemoradiotherapy^15^. In contrast, a study performed by Hou *et al.* suggested that *PIK3CA* mutation is associated with a significantly longer overall survival in patients with metastatic or recurrent cervical squamous cell carcinoma^10^. Wright *et al.* reported that PIK3CA mutations were associated with shorter survival in unspecified cervical cancers^14^. In our study of a large cohort of Chinese patients who underwent surgery-based multimodal therapy, *PIK3CA* mutations were associated with a longer RFS on both univariate analysis and multivariate analysis including almost all risk factors for recurrence (pathological subgroup, FIGO stage, lymph node metastasis, parametrial involvement, lymph vascular involvement and deep myometrial invasion).

There were some studies which addressed that *PIK3CA* mutation associated with treatment outcomes in cervical cancer^10^,^14^,^15^,^21^, however, the sample sizes were too small to draw a conclusion. In this study of a large cohort of Chinese patients who underwent surgery-based multimodal therapy, *PIK3CA* mutations were associated with a longer RFS on both univariate analysis and multivariate analysis including various risk factors for recurrence. On the contrary, *PIK3CA* mutation was reported to be associated with poor response or poor survival after definitive radiotherapy by McIntyre *et al.*^15^ and De la Rochefordiere *et al.*^21^. Furthermore, it had been observed that constitutively activated PI3K pathway promoted resistance to radiation and inhibitors of PI3K pathway radiosensitized human cervical cancer cell lines^22^,^23^. The reasons for the differences between our study and their studies were as follows: (1) all the tumors in this study were surgically resected cancers staged as FIGO IB1-IIA2, relatively earlier stage than the tumors treated with primary definitive radiotherapy in the other studies; (2) In this study, all the patients received primary radical surgeries and a considerable proportion of patients received adjuvant radiation after radical surgery. After radical surgery, there were no visible residual tumors in patient’s body, which would minimize the impact of *PIK3CA* mutation on radioresistance; (3) We observed higher risk of distant metastases after treatment in *PIK3CA* wild-type cohort (14.7%) than *PIK3CA* mutant cohort (7.6%) (p = 0.048). Although the reason for this is not clear, similar results have been reported in breast cancer. *Sabine et al.* reported that patients with breast cancer without *PIK3CA* mutations were at a significantly higher risk of distant metastases than patients whose tumors harbored *PIK3CA* mutation^24^.

This study contains certain limitations. First, given that HPV genotyping is not routinely analyzed for cervical cancer patient management, HPV genotyping analysis was not included in this study. Second, whether the rare mutant variants observed in our study could activate the *PI3K* pathway requires functional verification. Third, due to the relatively short follow-up period and the small proportion of the patients dying from cervical cancer, the overall survival (OS) was not calculated in this study. Furthermore, because of the limited number of cases with a mutation in exon 20, we were unable to compare clinical features between patients with *PIK3CA* mutations in exon 9 and patients with PIK3CA mutations in exon 20.

In summary, our data demonstrate that a considerable proportion of Chinese cervical cancer patients harbor mutant *PIK3CA*. Patients with mutant *PIK3CA* tumors show significant differences when compared with patients with *PIK3CA* wild-type tumors with respect to age, menopausal status, histological subtype, treatment outcome and recurrent pattern after surgery-based multimodal therapy.

## Methods

### Patients and specimens

This study was approved by the Ethics Committee of Fudan University Shanghai Cancer Center (FUSCC 050432-4-1212B) and was carried out in accordance with the approved guidelines. All patients provided written informed consent. In total, 771 Chinese women were included in this study. Each of them satisfied the following criteria: pathologically confirmed primary cervical carcinoma, FIGO stage IB1-IIA2 disease, no neoadjuvant chemotherapy or radiotherapy, having undergone radical surgery for the primary tumor, and no pre-operative conization. The tumor specimens were prepared and processed as previously described^8^. Cervical tumor specimens were collected during radical hysterectomy or trachelectomy procedures between January 2010 and December 2012. Histological assessment of the specimens was performed by two independent pathologists (Xuxia Shen and Wentao Yang), and only specimens with more than 50% tumor cells were included in our analysis. The tumor specimens were preserved in RNAlater solution (Ambion) and stored at −80 °C until analyzed. Clinicopathological data were prospectively retrieved from patient medical records. The patients were followed up regularly until relapse occurred.

### Mutation analysis

*PIK3CA* mutation analysis was conducted without prior knowledge of patient clinicopathological and follow-up data. Total RNA extracted from the tumor tissues was reverse-transcribed into single-stranded cDNA using an M-MLV reverse transcriptase kit (Invitrogen). The helical domain (HD) and the kinase domain (KD) of PIK3CA was PCR amplified with KOD-Plus-Neo DNA polymerase (Toyobo) using the following primers: HD-F: TTCAGCAGTGTGGTAAAGTTCC; HD-R: TACCAAGCAATACATCTGGGCTAC; KD-F: CTGGATACTGTGTAGCTACCTT; KD-R: ATGGATTGTGCAATTCCTATG. The PCR products were directly sequenced from both ends using Sanger sequencing. All mutations were confirmed by an additional independent PCR.

### Statistical analysis

The relevance between *PIK3CA* mutations and clinicopathological characteristics was analyzed by Student’s t-test, Pearson’s chi-square test or Fisher’s exact test. Relapse-free survival (RFS) was defined as the period of time from surgery to the first local, lymph node or distant recurrence. Distant metastasis was defined as any relapse outside of pelvic cavity. Patients who died without evidence of disease were censored. Due to the short duration of patient follow-up, the overall survival (OS) was not calculated in this study. RFS was assessed using the Kaplan–Meier method, and differences between the groups were analyzed using the log-rank test. Hazard ratios (HRs) were calculated using Cox proportional-hazards models. The two-sided significance level was set at P < 0.05. Data were analyzed using IBM SPSS Statistics 19 (IBM).

## Additional Information

**How to cite this article**: Xiang, L. *et al.* *PIK3CA* mutation analysis in Chinese patients with surgically resected cervical cancer. *Sci. Rep.* **5**, 14035; doi: 10.1038/srep14035 (2015).

## Supplementary Material

Supplementary Information

## Figures and Tables

**Table 1:** Nonsynonymous mutations identified in Chinese cervical cancer patients (No. = 105).

**Amino acid change**	**Nucleotide change**	**No. of cases**	**Frequency of variants (%)**	**COSMIC reported**	**Function**
p.E545K	c.1633G > A	59	7.7	Yes	Pathogenic
p.E542K	c.1624G > A	32	4.2	Yes	Pathogenic
p.H1047R	c.3140A > G	4	0.5	Yes	Pathogenic
p.M1043I	c.3129G > T	1	0.1	Yes	Pathogenic
p.E545A	c.1634A > C	1	0.1	Yes	Pathogenic
p.E545Q	c.1633G > C	1	0.1	Yes	Pathogenic
p.E545D	c.1635G > T	1	0.1	Yes	Pathogenic
p.H1047L	c.3140A > T	1	0.1	Yes	Pathogenic
p.Y1021C	c.3062A > G	1	0.1	Yes	Unknown
p.Y1021H	c.3061T > C	1	0.1	Yes	Unknown
p.G1049A	c.3146G > C	1	0.1	Yes	Unknown
p.R975K	c.2924G > A	1	0.1	No	Unknown
p.L523I	c.1567 T > A	1	0.1	No	Unknown

**Table 2:** Comparison of clinicopathological characteristics of cervical cancer patiens based on PIK3CA mutation status.

Clinicopathologic characteristics	Total number	PIK3CA mutation status		P value
		Wild-type (n = 666)	Mutant (n = 105)	
Age (years)				<0.001
Mean	47.5	47.0	50.7	
SD	9.3	9.2	9.7	
Menopausal status				<0.001
Premenopausal	491	441	50	
postmenopausal	280	225	55	
Histological subtypes				0.007*
SCCs	606	513	93	
ACs	101	92	9	
ASCs	44	43	1	
Others	20	18	2	
FIGO stage				0.328
IB	394	345	49	
IIA	377	321	56	
Node status				0.907
Negative	569	492	77	
Positive	202	174	28	
Tumor sizes				0.735
>4 cm	217	186	31	
≤4 cm	554	480	74	
Depth of myometrial invasion				0.100
whole-thickness	280	241	39	
>1/2	252	210	42	
≤1/2	239	215	24	
LVSI				0.830
Yes	305	262	43	
No	466	404	62	
Parametrial involvement				0.255
Yes	44	41	3	
No	727	625	102	
Relapse				0.022
Yes	142	131	11	
No	629	535	94	
Death				0.029
yes	86	81	5	
no	685	585	100	

*compared between SCC with non-SCC subtypes.

SCC: squamous cell carcinomas; AC: adenocarcinomas; ASC: adenosquamous carcinomas; LVSI: lymph vascular Involvement.

**Table 3:** PIK3CA mutations in cervical cancer predicted independently better RFS in univariate and multivariate analyses.

variables	**Univariate analyses**			**Multivariate analyses**		
	**HR**	**95% CI**	**P-Value**	**HR**	**95% CI**	**P-Value**
Age(>50 years)	0.94	0.67–1.32	0.725	–	–	–
Postmenopausal	1.11	0.79–1.56	0.540	–	–	–
Node status	3.32	2.39–4.62	<0.001	1.67	1.11–2.50	0.013
Depth of myometrial invasion	2.15	1.71–2.71	<0.001	1.51	1.15–1.97	0.003
Parametrial involvement	4.63	3.00–7.14	<0.001	2.18	1.36–3.50	0.001
Tumor sizes	1.65	1.17–2.32	0.004	1.29	0.91–1.83	0.147
LVSI	2.66	1.90–3.72	<0.001	1.49	0.99–2.22	0.053
FIGO stage	1.94	1.73–2.38	<0.001	1.51	1.05–2.17	0.025
Histological subtypes*	2.06	1.44–2.95	<0.001	2.43	1.68–3.50	<0.001
PIK3CA mutation status	0.51	0.28–0.95	0.033	0.54	0.29–0.99	0.048

*compared non-SCC subtypes with SCC.

SCC: squamous cell carcinoma; LVSI: lymph vascular Involvement.
