# Supplementary material for: PIK3CA mutation analysis in Chinese patients with surgically resected cervical cancer
Source: Sci Rep. 2015 Sep 11;5:14035. doi: 10.1038/srep14035 (PMC4566086; doi:10.1038/srep14035)
Supplement: Supplementary Information [file srep14035-s1.pdf]

PIK3CA mutation analysis in Chinese patients with surgically resected cervical cancer

Libing Xiang[1]， Wei Jiang[1], Jiajia Li[1], Xuxia Shen[2], Wentao Yang[2], Gong Yang*[3], Xiaohua Wu*[1], Huijuan Yang*[1]

[1] Department of Gynecological Oncology and [2] Department of Pathology, Fudan University Shanghai Cancer Center, Department of Oncology, Shanghai Medical College, Fudan University, Shanghai, 200032, China. [3]Cancer Institute, Fudan University Shanghai Cancer Center, Department of Oncology, Fudan University Shanghai Medical College, Shanghai, 200032, China, Central Laboratory, The Fifth People's Hospital of Shanghai, Fudan University, Shanghai 200240, China.

Supplementary figure Legends:

Supplementary figure S1. Relapse-free survival (RFS) according to PIK3CA mutation status in patients with non-squamous subtypes of cervical cancer (no-SCC) (A) and in patients without adjuvant therapy after surgery (B).

Supplementary figure S2. Relapse-free survival (RFS) according to specific PIK3CA mutations on different sites of amino acid residuals(A) and on different exons (B).

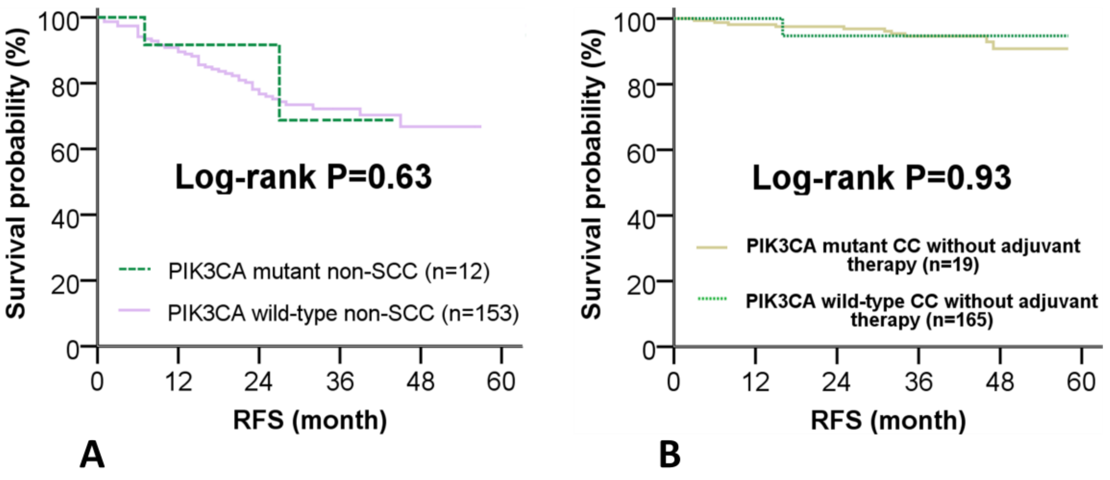

Supplementary figure S1.

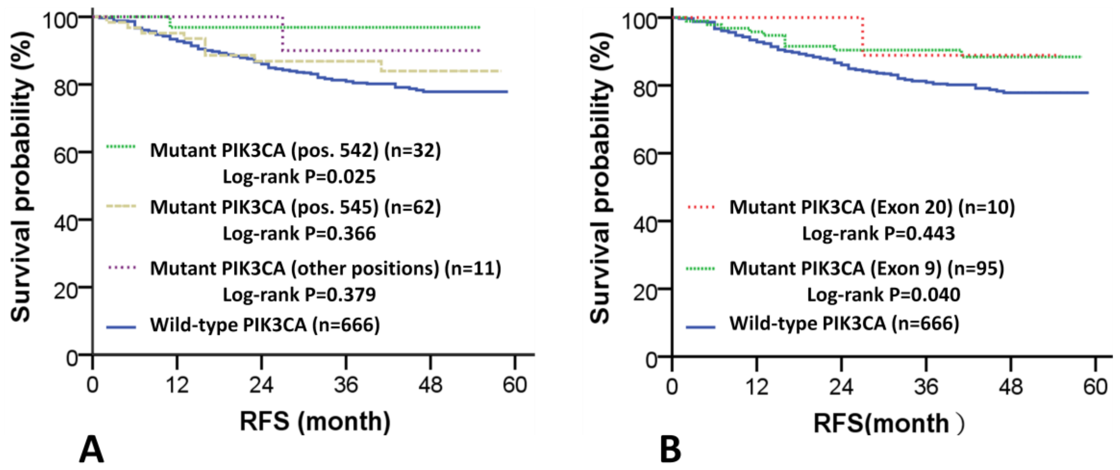

Supplementary figure S2.
